# Relatives’ grief at three moments after death of a loved one during COVID-19 pandemic (the CO-LIVE study)

**DOI:** 10.1080/07481187.2023.2297055

**Published:** 2023-12-25

**Authors:** Corine A. Nierop-van Baalen, F. Erica Witkamp, Ida J. Korfage, H. Roeline Pasman, Yvonne N. Becqué, Masha S. Zee, Agnes van der Heide, Bregje D. Onwuteaka-hilipsen, Anne Goossensen

**Affiliations:** a Research Centre Innovations in Care, Rotterdam University of Applied Sciences, Rotterdam, The Netherlands; b Department of Public Health, Erasmus MC, University Medical Center Rotterdam, Rotterdam, The Netherlands; c Department of Public and Occupational Health, Expertise Center for Palliative Care, Amsterdam UMC, VU University, Amsterdam, The Netherlands; d University of Humanistic Studies, Utrecht, The Netherlands

## Abstract

COVID-19 has complicated grieving experiences. Rich qualitative description of these experiences is lacking. We interviewed 10 bereaved relatives (mainly daughters) 2–3 times each: shortly after their relative died in the first wave of COVID-19 pandemic, and after 12 and 18 months (29 interviews in total). Analyses took place according to inductive content analysis. Losses were threefold: the loss of the loved one; of the (desired) way to say farewell, and of social support. We identified five ways in which the three COVID-19 related loss experiences interacted: overshadowed grief, cumulative grief, triggered grief, derailed grief, and conciliatory grief. This study demonstrated that pre-COVID-19 diagnoses and understandings of grief are not sufficient to picture grief during and after the COVID-19 pandemic. These grief experiences are more complex and deserve further exploration.

## Introduction

It is becoming increasingly clear what a devastating impact the COVID-19 pandemic and associated measures have had on people who lost a loved one (Hanna et al., 2021; Myers & Donley, 2022; Wang et al., 2022). Understanding the grief of bereaved relatives is an ongoing and important theme related to the COVID-19 crisis. The lack of access to and physical contact with relatives at the time of death and restrictions regarding funerals were highly distressing for bereaved relatives (Hanna et al., 2021; Onwuteaka-Philipsen et al., 2021), with potential long-term impacts on the grieving process (Neimeyer & Lee, 2022; Burrell & Selman, 2022; Hanna et al., 2021; Lee & Neimeyer, 2022; Pearce et al., 2021).

The studies that exist on the impact of COVID-19 pandemic on grief and bereavement demonstrate traumatic death experiences, restricted memorialization practices, and contending with the ongoing threat of the virus, as well as societal responses to the pandemic (Torrens-Burton et al., 2022). Portuguese bereaved adults present more anxiety and depression symptoms due to COVID-19 pandemic and the public health measures (Aguiar et al., 2022). Turkish bereaved relatives felt remorseful as they were not able to see or bring their loved one home during their last moments and felt deprived of the traditional rituals (Patel et al., 2022).

Eisma and Tamminga (2020) showed that adults who had recently lost a family member experienced more grief than those with recent loss before the COVID-19 pandemic. Von Blanckenburg et al. (2023) demonstrated that, during the COVID-19 pandemic, bereaved relatives had increased risk of prolonged grief when compared to before the pandemic. Visitor restrictions and being unable to say farewell at the death bed were found to be a risk factor for prolonged grief (Von Blanckenburg et al., 2023) or complicated grief (Gesi et al., 2020). It appears that the circumstances in which the loss occurred during the COVID-19 pandemic hindered the grieving process of the relatives in different ways (Mortazavi et al., 2020; Wallace et al., 2020). Gesi et al. (2020) demonstrated that poor communication with physicians during hospitalization increased the risk of complicated grief. Goveas and Shear (2020) described the circumstances of the death, as people dying alone; the context of the death, such as physical distancing policies and the consequences of the death, such as being alone as risk factors for prolonged grief disorder. Other challenges that were faced by persons whose relative had died during the pandemic were unfinished business between the bereaved and the deceased (Becqué et al., 2023; Holland et al., 2020). Unfinished business means incomplete, unexpected, or unresolved issues, for example, unfulfilled wishes or missed opportunities (Klingspon et al., 2015). Becqué et al. (2023) found that a negative appreciation of the dying process and remaining unfulfilled wishes as part of “unfinished business” between the dying person and their relative were associated with higher levels of despair, particularly among partners.

It is generally believed that the circumstances of the death, such as the place of death, influence the complexity of grieving processes (Stroebe & Schut, 2021). In case of the COVID-19 pandemic, these circumstances were negatively influenced by social distancing, isolation, uncertainty and self-blame regarding the infection, and inability to have the usual burials or funeral ceremonies. An integrative review on the views of bereaved families on the death of loved ones due to COVID-19 (Firouzkouhi et al., 2021) classified circumstances into two categories, before and after death. The circumstances before the death of the loved ones were described as lack of visiting options, absence at death time, fear of being infected with the COVID-19, inability to perform religious rites at death, and psychological problems. The after-death issues were related to funeral and burial rituals, prolonged grieving, maladaptation, loneliness, and serial losses. Having to experience that a family member dies alone may lead to feelings of guilt and abandonment in bereaved relatives, which can result in complicated grief (Boelen & Smid, 2017; Morris et al., 2020a). In complicated grief, painful emotions are more severe and last longer than in “normal” grief (Shear, 2022). Previous pandemics such as Ebola appear to have also caused a disruption to social norms, rituals, and mourning practices (Gesi et al., 2020; Mayland et al., 2020). This affects an individual’s ability to interacting with the deceased both before and after increasing the risk of complicated grief (Mayland et al., 2020) and impacts their overall death, potentially wellbeing (Hamid & Jahangir, 2022).

It is generally established that, for most individuals, bereavement lasts between several months and several years (Bergeron, 2023; Bonanno, 2019). Bereavement, the journey associated with the experience of loss and grief, is an individualized one (Mitchell, 2022) and the length is far from predictable (Bonanno, 2019). Although grief has been extensively studied in the literature, much less is known about the duration of the bereavement process, which appears to be much more individual and dependent on a person’s resilience (Coifman et al., 2007). Moreover, bereavement research has typically centered on one time point, without considering the impact over time (Schwartz et al., 2018). Most studies of bereavement assess the course of grief on a single response to loss without considering the multiple extrinsic circumstances surrounding the death (i.e., relationship to the deceased, type of death, time since loss, etc.; Bonanno et al., 2002; Mancini & Bonanno, 2010).

Overall, however, there remains a lack of knowledge on the many different and in-depth grief experiences of relatives during COVID-19 pandemic over time. The COVID-19 epidemic is unlikely to be the last epidemic in the world, we therefore want to better understand the impact of this episode on the grieving process over a longer period. The aim of this study was to get in-depth and longitudinal insight into the bereavement of relatives who lost a loved one during the COVID-19 pandemic.

## Methods

To gain insight into the bereavement of relatives during the COVID-19 pandemic, we chose a qualitative design involving in-depth interviews. We used an inductive content analysis design to understand the bereavement experiences.

### Data recruitment and collection

Via professional networks and public campaigns, potential participants were referred to a national website, where they could find information about the CO-LIVE study and where they were invited to complete a questionnaire. Participants for this interview study were recruited from those who completed questionnaires and indicated a willingness to be interviewed. Through purposive sampling, we selected participants with diverse backgrounds in terms of gender, age, and setting where the person died. Interviews took place between May 2020 and September 2021. This study is embedded in a national open online questionnaire survey of experiences during the COVID-19 pandemic in the Netherlands: The CO-LIVE study (Yildiz et al., 2022).

Individual in-depth interviews were conducted to gain an understanding of the grieving experiences of bereaved relatives. Due to the COVID-19 pandemic the interviews were conducted online via Zoom and audio recorded. The interviews were conducted in the Dutch language. The interviews were loosely structured using a topic guide. Topics discussed were the impact of COVID-19 related to the circumstances of saying farewell, after-death experiences, funeral, relatives’ grief, and the bereavement process. Interviewees had the opportunity to discuss experiences they additionally considered relevant. We conducted interviews with 10 bereaved relatives at baseline (shortly after completion of the first questionnaire) after 12 months, and after 18 months. Table 1 demonstrates the demographic characteristics of the respondents and their relationship to the deceased. All interviews were conducted by the same project group member [AG], who is well acquainted in conducting interviews on sensitive topics, such as dying and mourning, and in qualitative research. Interviews were transcribed verbatim.

**Table 1.** Demographical characteristics of the respondents.

Interviewee	Relationship to the deceased	Gender and age of the deceased	Place of death
1	daughter	Woman, 84 years	Hospital
2	wife	Man, 74 years	Hospital
3	husband	Woman, 73 years	Hospital
4	daughter	Man, 83 years	At home
5	daughter	Man, 74 years	Hospital
6	wife	Man, 75 years	Nursing home
7	daughter	Man, 89 years	Hospital
8	Daughter	Man, 91 years Woman, 89 years	Nursing home
9	Daughter	Woman, 95 years	Nursing home
10	Daughter	Woman, 88 years	Hospital

### Data analysis

This study used inductive content analysis (Kyngäs, 2020) to explore the grief experiences after losses that happened during COVID-19 restrictions. We followed a phenomenologically inspired way of analyzing the material, meaning that we focused on the description of how bereavement manifested itself during the day, in the body, emotionally, relationally, and in meaning making (Dahlberg & Dahlberg, 2020).

Several measures were taken to increase the rigor of the study. To address trustworthiness, we were inspired by Lincoln’s and Guba’s (1989) criteria. Concerning credibility, the analyses were facilitated by the last author’s familiarity with the field of dying at the time of COVID-19, the restrictions, and grieving experiences. Through extensive discussions with two other researchers well known in the field of dying and bereavement, both as researchers and as health professionals, dependability and confirmability were enhanced. Authenticity also was enhanced by researcher triangulation and by considering the social context of the participants.

The first author read all the transcripts in full to acquire an overall picture of what had been discussed, and subsequently read through again to grasp in more detail what had been said about bereavement in times of COVID-19. All transcripts from each participant were analyzed as a series of interviews of three per person to investigate individual experiences and changes over time. The interviews were analyzed employing constant comparative analysis as adapted by Boeije (2009). Each passage in the interviews was assigned a code to represents its content and the initial coding was fully inductive. After this open coding, we integrated the codes around themes through axial coding. Two researchers listened to all the interviews and/or read the transcripts, and read each other’s analysis notes, commented on them, and discussed each other’s interpretations. Then, emerging themes were identified and developed by studying the transcripts and the codes and by considering possible meanings and the way they are connected and distinguished. Finally, by selective coding, the themes were verified, further refined, and developed. This process, the codes and emerging themes were carefully discussed with three other researchers (one of whom conducted all the interviews). The findings about the triple losses emerged in these reflection meetings. The first author subsequently consulted the data again to verify the interpretations against the data. The prominence of experiences of bereavement in the interviews allowed for a rich description of bereavement during COVID-19 pandemic. The quotes and results were then translated into English and discussed several times with the team of researchers and modified to best reflect these findings.

### Ethical considerations

Ethical approval was obtained from the Erasmus MC Medical Ethical Committee [ref. MEC-2020–0254]. All respondents were given online and oral information about the study. Participants provided oral informed consent, which was audiotaped and transcribed verbatim. Pseudonymization and confidentiality were guaranteed, and respondents were informed that they could withdraw from the study at any time. The interviewer aimed to be attentive to the respondents’ grief and to create a comforting atmosphere. Some respondents had had little opportunity to talk about their grief-related emotions. In these cases, the interviewer encouraged them to find someone with whom to disclose their experiences.

## Results

A total of 29 interviews with 10 relatives were analyzed; one of the interviewees was unable to participate in the third interview due to severe mental stress. Most of the respondents were daughters of the deceased. The analysis revealed that the grief experience of the respondents was essentially an interaction of triple losses. How these losses affected their lives differed. Respondents showed different (and interacting) grief responses to these losses that emerged from the COVID-19 pandemic: overshadowed grief, cumulative grief, triggered grief, derailed grief, and conciliatory grief.

### Triple loss

All respondents experienced a triple loss. This means that they had lost their loved one, they had lost the (desired) opportunity to say goodbye according to their own values, and they lost social support for the respondents due to the COVID-19 measures. A loss that had a lot of impact was experienced when they were not able to have any form of contact with their loved ones at the time they were seriously ill. This loss made the dying process felt unfinished and left relatives with the feeling that they were not able to be there for their loved one at important moments, while respondents considered this closeness to be very important and necessary for their loved one and for themselves at that moment.

She died on 20 May, and from 20 March onwards, I think this visiting ban had been imposed. So, the last seven, eight weeks of her life, when she was still well, relatively well, which included her birthday and Mother’s Day, etc., it was difficult for us to do anything at all there, no visits or anything like that. […] That was the most terrible thing, that you could not be there for her, while you really needed to be. (daughter of mother in nursing home)

In addition to the loss of their loved one, the relatives also experienced the loss of the desired manner of saying goodbye. For some, saying goodbye was not allowed at all, or with a limited number of people, for example a maximum of two. This meant that some daughters did not see their parent before or even after death, when a ceremony was not possible. For others, their parent had clear wishes in the event of their death, and the children were unable to carry these out due to the corona measures. This not only caused the children to feel pained that they have not been able to carry out their parent’s wishes, it also hurt their dignity values.

That we couldn’t wash and dress him properly, that he was just buried in his old T-shirt. And that still makes me sad, that remains there. […] But the fact that you really just could not carry out his last will, that remains difficult. At least for your own processing. (daughter of father who died in hospital)

The respondents also experienced a loss of social support in their grief and mourning. On the one hand, because often the funeral or cremation was only possible with a limited group. This meant that with large families, choices had to be made about who could or could not attend the funeral. This also always meant that people who wanted to come were not given the opportunity to do so. On the other hand, there were important family members or loved ones who could not come to the funeral because they were either infected with COVID themselves, and sometimes turned out to be hospitalized, or were in isolation at home or did not dare to come because of their own fragile health The loss of social support had an impact on relatives’ grief experiences. Shortly after the funeral and in the period that followed, social support was also lacking. Literally sitting at home and not receiving any visitors made people feel very lonely.

You know, there were rules in those days. You were only allowed 30 people in a church. Our family alone consists of 39 people, including children and grandchildren. So, then you have a lot more [persons] than the siblings. […] After that funeral, each of the children went to their own house. You could never be all together, just never. (wife of husband who died in hospital)

These circumstances of death, limitations in saying goodbye and isolation had a great impact on the grief of loved ones and this impact lasted for a long time, perhaps forever.

And I do have a very strong feeling, of course, that those last two months we didn’t have with my mother […], you can never catch up with that. That’s why I don’t say I'm angry about it, that there is anger in it. But it is because of what you have been through that you do experience the impact of it. And I think I will always keep that with me. (daughter of deceased mother)

### Experiences of bereavement

The experiences of bereavement showed the different reactions of bereaved people to the threefold losses. These reactions of the respondents reflect the diversity as experienced by them during the COVID-19 pandemic. We identified five interacting grief’ processes arising from the COVID-19 pandemic: overshadowed grief, cumulative grief, triggered grief, derailed grief, and conciliatory grief.

### Overshadowed grief

In overshadowed grief, the mourning for the loss of the deceased loved one is overshadowed by the pain about the circumstances in which this death took place.

Yes, what I notice above all is that the circumstances have just shifted the mourning a bit, that - now comes the loss and the mourning that I would normally have experienced a bit earlier, I think. Just because of everything that happened around it, the whole strange situation, that it just took a long time before the real mourning process started. (daughter of deceased parent in nursing home)

Overshadowed grief also considered mourning missed opportunities. These relate to circumstances where in hindsight, more would have been possible, in the sense of longer visiting hours, more people visiting or parents who in hindsight could have been together after all. Mourning over missed opportunities meant that grief about the person(s) who died is overshadowed by thoughts that things could have gone differently, and therefore more pleasantly or less badly.

And then afterwards the nurses said: your father could have stayed in his room. Because we have several couples lying here together. [So I thought, sh*t, they could still be together. And still would have been able to die together. And that is what I find very sad. And also because you didn’t get to say goodbye to your father. (daughter of deceased parents; one died in hospital, the other in a nursing home)

Overshadowed grief means that relatives still struggled with the lack of goodbye, and this affects the pain of the loss and the circumstances in which this took place, as well as getting used to and dealing with the new situation of picking up life again. Overshadowed grief was also about (having to) live in the COVID-19 pandemic and with the associated measures. Because the COVID-19 pandemic had such an impact on everything and everyone in daily life, some people only realized after measures have been relaxed and normal life had restarted that the death of their loved one also meant that they had to miss them.

And I found it especially difficult when things eased up a bit and more was possible again, when normal life got going. Then I actually suffered a bit of a setback. It was as if I only started missing my father then. (daughter)

### Cumulative grief

During their mourning process, some relatives were confronted with new life events. These often concerned other family members falling seriously ill or new deaths due to COVID-19. The new losses often involved again a triple loss, of their loved one, the lack of contact with the seriously ill, and in some cases another farewell. All this made the losses compound and grief became very complex. The experiences of cumulative losses and thus cumulative grief made one feel crushed under the pile of life events that interacted and aggravated the emotional wounds.

So much has happened in my life in the meantime that I just can’t pick up the thread. Well, our son was ill then too. He also had corona. And he can do almost nothing. His wife is burnt out now. Really very bad. I have a sister-in-law who committed suicide. And I have a brother-in-law who died of corona. And then you have to pick up your life. (wife)

In cumulative grief we saw that the grief and misery were too great to be manageable. Relatives did not oversee this grief and had no idea how, where, and when to start to be able to bear these losses. Relatives felt that their ‘bucket’ was already very full and was in danger of overflowing: “It is quite something to lose two parents in five days.”

### Socially triggered grief

Socially triggered grief was about mourning, the pain of loss, which could unexpectedly and suddenly flare up due to COVID-19 pandemic situations. In this grieving process, for example, subsequent lockdown periods and discussions about the measures appeared to act as triggers for the grieving bereaved, who thus saw their grief (temporarily) exacerbated. The constant reports in the media could also lead to a flare-up of their pain, grief, and intense memories.

It’s just that you were constantly reminded of it because of the ongoing process. And sometimes I had trouble with that. Certainly, if you then happened to see a piece on television again, (cries). Then I sometimes want to be thrown back to a year ago. (husband)

Also, when relatives heard that a close acquaintance or family member had COVID, they felt thrown back into the situation and into their intense grief. The fact that not all friends and acquaintances were able to say goodbye and therefore relatives could often only complete the saying goodbye when they met with their friends or acquaintances (months) later, made relatives feel that the period of intense grief lasted longer.

But what I still struggle with now is that there are many acquaintances who have not been able to say goodbye personally or offer condolences. And now I keep running into acquaintances who look at me and say oh, good to see you. So, I'm still mourning. (husband)

Another form of triggered grief showed itself in/as anger directed at others who did not adhere to the corona-related measures or who were unvaccinated.

I was very annoyed by the corona deniers last year. That bothered me a lot. […] last year I entered into fierce discussions with someone […] But yes, I said, well, I'm not going to do it anymore. Because then I take it home again and then, eh, I have a bad day and they just cycle on. So there [laughs], I've got rid of that. (husband)

Socially triggered grief was about other relatives’ opinions on whether or not people living in a nursing home should receive visits. These opinions of others could rekindle one’s own grief about missed and cherished moments of contact, and exacerbated this grief and the pain associated with it.

### Derailed grief

Sometimes grief took extreme forms, such that it threatened to derail. This was the case when the circumstances of the death of a loved one had a traumatic effect on the relatives. This traumatic way could express itself in flashbacks, nightmares, and other intense symptoms, such as fears, which required Eye Movement Desensitization and Reprocessing (EMDR) treatment. EMDR treatment turned out to be necessary because, for example, the respondents still had all kinds of reeexperiences about the sounds of the corona ward where the patient stayed until the end of his or her life. Sounds such as screaming, crying, and beeping infusion pumps haunted the respondents for a long time and severely hampered his or her ability to resume life. In original grief, grief and past experiences became more intense instead of milder. People also developed physical complaints or made existing complaints worse. Some also developed fears, for example not daring to go into a shop. One daughter of deceased parents asserted, "I had nightmares and couldn’t sleep. And I kept reliving all those images in the nights. And they got worse and worse.” Others experienced extreme difficulty to handle grief because of the impact of the experience surrounding the death. This was the case of the daughter who lost her father to suicide: “It’s all so horribly awful”.

### Conciliatory grief

In conciliatory grief, relatives described that they were at peace that their loved one had been spared further suffering due to illness or circumstances. Reconciliatory grief was only seen among respondents whose loved one was already seriously ill, for instance as due to dementia, chronic obstructive pulmonary disease, or a hematology-oncology condition. Reconciliation was expressed by being at peace with the death and relief that their loved one did not have to deal with further social isolation and loneliness.

And that [hematologist] said, “Mr. V, I think your wife- I'm not allowed to say it like that, but she’s better off this way. Because the leukemia had progressed. So, she would have had a very bad year. And in the end, she would have died.” So that’s a small consolation. (husband)

Reconciliation was also expressed in that people could look back with satisfaction on the care, medical treatment and pleasant conversations with doctors and nurses. People experienced the feeling of being in good hands. One husband of a wife who died in hospital explained, "But they [the doctors at the hospital] were really on top of it. That was reassuring, also for my wife. Because she slept well there, she knew she was in good hands really." Another form of reconciliation was seen in relatives who actively attributed a positive meaning to the loss in terms of that further suffering was spared. A daughter of deceased parents described, “But it is also a comfort that they are together now, isn’t it? That they didn’t have to leave each other behind. So, then we try to do it right in that way."

## Discussion

Our analysis of 29 interviews with 10 bereaved families during the COVID-19 pandemic revealed the enormous impact and the different reactions of bereaved people to the three losses – the loss of their loved one, the loss of the desired way to say farewell, and the loss of social support due to the COVID-19 measures. Coping with different and often simultaneous losses is expressed in the grief experience. Overshadowed grief described the struggle of people with the lack of saying goodbye and the circumstances in which this took place, and this affects the pain of the loss, as well as getting used to and dealing with the new situation of picking up life again. In cumulative grief the grief and misery were too great to be manageable. People did not oversee this grief and had no idea how, where, and when to start to be able to bear these losses. Triggered grief was about mourning, the pain of loss, which could unexpectedly and suddenly exacerbated due to COVID-19 pandemic situations from outside. The constant reports in the media could also lead to a flare-up of their pain, grief, and intense memories. In derailed grief, the grief sometimes took extreme forms, such that it threatened to derail. This was the case when the circumstances of the death of a loved one had such an impact that it had a traumatic effect on the family. In conciliatory grief, the loved ones were at peace that their loved one had been spared further suffering due to illness or circumstance or reconciliation was seen in respondents who actively attributed a positive meaning to the loss and were able to derive comfort from it. The different aspects of the loss affected relatives’ grieving experiences in different ways.

Our respondents experienced a major impact of not being present at the time of death and not being able to say goodbye to their loved one. Dew et al. (2022) also demonstrated that not being able to be present at the time of death made it more difficult to accept the death of a loved one. Performing religious ceremonies, attending at the patient’s bedside at the time of death has been impossible for COVID-19 patients (Ahaddour et al., 2017), which resulted in increased grief in families (Romero et al., 2014). Otani et al. (2017) explored the potential association between the family’s depression and complicated grief and their presence at the moment of a patient’s death and the patient’s communication with the family. They concluded that many families wished to be present at the moment of the patient’s death. However, not the presence or absence at the moment of death, but meaningful communication (saying ‘‘goodbye’’) between a patient and family members before the former’s death was associated with better outcomes on measures of depression or complicated grief.

Our findings demonstrated that all respondents experienced a triple loss, existing of the loss of the loved one, the (desired) way to say farewell and of social support. Schwartz et al. (2018) found that participants who had experienced two losses endorsed significantly more grief symptoms than those with a single loss. They conclude that multiple losses impact grief and create more vulnerability to complex grief processes. Our findings about the five typologies of bereavement indicate that the existing bereavement models, which mainly focus on single losses could be further developed and ameliorated. Further research could focus on to what extent and in what way the different grief processes following the multiple losses interact with each other. And what is helpful for recovery.

Our results align with the findings of Becqué et al. (2023), who found an association between "unfulfilled wishes" regarding the loved one and despair during grief. Our findings indicate that many respondents had to deal with “unfinished business” due to the restrictions, where some were not allowed to say goodbye or they could not fulfill the wishes surrounding the farewell. Higher unfinished business scores were previously associated with more negative grief symptoms (Klingspon et al., 2015; Lobb et al., 2010). Additionally, our findings showed that the threefold losses and the different bereavement experiences followed a different time course. Our results suggest that these processes do not run parallel in time, can differ per loss, and seem to be (partly) related to external circumstances. This might raise topics for further development of supporting bereaved people. Now that worldwide almost seven million COVID-19 deaths have been registered (WHO, 2023), there is an urgent need for more differentiated grief models, considering the interaction of the multiple losses the bereaved have experienced.

Our study emphasized the impact of the circumstances after death, caused by the social restrictions. Fuller et al. (2021) stated that during COVID-19 pandemic it was often impossible to organize the desired funeral, which caused psychological problems in the families and remained a bitter memory in their mind. According to Firouzkouhi et al. (2021) religious rites can make death acceptance easier and the suffering of losing loved ones more bearable. Being able to practice death rituals in a pandemic environment also allowed people to express their feelings and adapted more quickly to the death of loved ones (Fernández & González-González, 2022; Myers & Donley, 2022). In addition, bereaved people had less opportunity to communicate with others or participate in the ceremony due to social distance and corona measures (Mortazavi et al., 2021; Morris et al., 2020b).

Although the circumstances of the grief appear to be unique to the COVID-19 pandemic, we also saw similarities with previous pandemics, such as the Ebola outbreak in West Africa (Mayland et al., 2020). Physical separation and distancing during earlier outbreaks exacerbated feelings of grief, loss, misery, guilt, and helplessness among family members and increase the risk for mental health problems (Aguiar et al., 2022). Our data revealed that sometimes grief took extreme forms, especially when the circumstances of the death of the loved one had such an impact on the family member, that it required EMDR treatment.

Torrens-Burton et al. (2022) analyzed qualitative data of bereaved relatives by inductive thematic analysis and then applying Stroebe and Schut’s Dual Process Model (DPM) as an analytic lens to further contextualize and interpret the data. Torrens-Burton et al. (2022) described examples of loss-oriented stressors included being unable to visit and say goodbye at the end of life and restricted funeral and memorialization practices. As in our results, they showed that severely curtailed support systems and reduced social/recreational activities have further undermined people’s current ability to cope with and adapt to bereavement.

Due to the COVID-19 pandemic, people experienced that there were missed opportunities, such as no opportunity to be with their loved one at the time of death. Our data showed the struggle people have with these missed opportunities. However, also in general people often struggle with missed opportunities in case of the death of a loved one. In other words, this finding is not new, but the frequency with which the various missed opportunities presented themselves is novel. A study by Witkamp et al. (2015a) showed that family members could experience feelings of guilt even if they were sitting next to the bed. Experienced missed opportunities appeared to be an important theme in grief. Another study by Witkamp et al. (2015b) conducted in a hospital setting showed that 77% of people died in close proximity to a loved one and that death close proximity of a loved one was considered very valuable.

This study has some limitations. This is an in-depth study, focusing on very sensitive experiences within family relationships of a small number of participants. For this analysis we only interviewed one husband, one wife, and mainly daughters at three different time intervals. However, despite the small scale of the study, the themes were clearly expressed. The sample was self-selected, we depended on the people who chose to be interviewed and followed people with more intense experiences during a longer period. It could be that people who like to share their experiences choose to participate. However, the richness and variety of the data collected at three intervals enabled in-depth analyses despite these limitations. Moreover, all the study participants had lost an elderly (> 75 years) relative or partner, and therefore our findings cannot be generalized to people whose younger loved ones died during COVID-19. The interviews all were performed online due to the COVID-19 restrictions. The differences in quality of online interviewing versus face-to-face interviewing are still undefined.

The strength of this study was that it examined in depth the experiences of grief and the challenges people faced regarding these losses, during COVID-19 pandemic. All the interviews were conducted by same researcher, who is well acquainted in conducting interviews on sensitive topics, such as dying and bereavement, and in qualitative research. she was also able to be attentive to the respondents’ situation and to create a comforting atmosphere, in which people could tell their story and feelings open-hearted.

## Conclusions

Relatives who lost a loved one during COVID-19 pandemic do not only suffer from losing their loved ones. They also lost the (desired) opportunity to say farewell and the social support due to the corona measures. These circumstances of death and having to say goodbye expressed itself in the different experiences of bereavement. We identified five forms: Overshadowed grief, cumulative grief, socially triggered grief, derailed grief, and conciliatory grief and described their characteristics. This study demonstrates that current understandings of grief are not sufficient to understand grief during and resulting of the COVID-19 pandemic. These grief experiences are complex and consist of several interacting grieving processes in response to the various losses. Further exploration is recommended of long-term recovery processes and of what are helpful elements in bereavement coaching and treatment.
